# Heteromultivalent Nanogels as Highly Potent Inhibitors of *Pseudomonas Aeruginosa*

**DOI:** 10.1002/anie.202513121

**Published:** 2025-11-11

**Authors:** Yuhang Jiang, Chuanxiong Nie, Boyu Zheng, Vinod Khatri, Denis Puccio, Yanping Long, Mathias Dimde, Rainer Haag, Sumati Bhatia

**Affiliations:** ^1^ Institute for Chemistry and Biochemistry Freie Universität Berlin Takustraße 3 14195 Berlin Germany; ^2^ Department of Chemistry Faculty of Science and Engineering Swansea University Swansea SA2 8PP UK; ^3^ Department of Chemistry TDL Govt. College for Women Murthal Sonipat 131027 India

**Keywords:** Bacteria inactivation, Biofilm dispersion, Heteromultivalent nanogels, Pseudomonas aeruginosa

## Abstract

The increasing prevalence of microbial resistance requires new antibacterial concepts for selective targeting and killing of pathogenic bacteria. Here, we report the synthesis of a heteromultivalent nanogel system against *Pseudomonas aeruginosa* (*P. aeruginosa*). These nanogels are based on biocompatible polyglycerols and functionalized with sugar ligands fucose (Fuc) or galactose (Gal) for *P. aeruginosa* targeting. With a further modification of these nanogels with BMAP‐18 short chain peptides (GRFKRFRKKFKKLFKKLS), we have achieved > 99.99% inactivation of planktonic and > 99.9% inactivation of biofilm‐coated *P. aeruginosa* within 12 h of treatment. Additionally, the system demonstrates broad‐spectrum antimicrobial potential, effectively inhibiting *Escherichia coli* (*E. coli*) and Methicillin‐resistant *Staphylococcus aureus* (MRSA). This modular design offers a promising strategy for the development of next‐generation antimicrobial therapies targeting biofilm‐associated infections and MDR bacteria.

## Graphical abstract

A heteromultivalent polyglycerol nanogel system integrates fucose/galactose and short‐chain antibacterial peptides to achieve strong bactericidal and anti‐biofilm effects at low concentration by efficient multivalent binding with bacterial lectins.

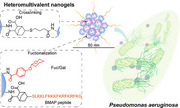

*Pseudomonas aeruginosa* (*P. aeruginosa*), a multidrug‐resistant (MDR) bacteria, was recognized as one of the most life‐threatening bacteria.^[^ ^1^, ^2^ ^]^ *P. aeruginosa* commonly causes nosocomial infections, which are fatal in immunocompromised patients due to its adaptability and high intrinsic antibiotic resistance.^[^ ^3^, ^4^ ^]^ *P. aeruginosa* produces biofilm and develops resistance against current clinical therapies using antibiotics. The scarcity of new antibiotics and the rapid development of antimicrobial resistance mark the need for alternative therapies against MDR *P. aeruginosa*.

The soluble proteins LecA (PA‐IL) and LecB (PA‐IIL) on *P. aeruginosa*, can be used as the target for inhibitor design, which specifically bind galactose and fucose, respectively.^[^ ^5^, ^6^ ^]^ Specific binding to oligosaccharides or glycoconjugates on the host cell surface by *P. aeruginosa* can be reduced through galactose/fucose‐lectin interactions, thereby inhibiting adhesion and interfering with effective cell‐to‐cell communication within the microbial community to reduce biofilm formation.^[^ ^7^, ^8^, ^9^ ^]^ However, the monovalent binding is not sufficient to significantly reduce virulence and clear infection.^[^ ^9^, ^10^ ^]^ Single sugar ligands require significant modifications to achieve satisfactory levels of antimicrobial potency and in vivo metabolism.^[^ ^11^, ^12^ ^]^ In addition, antimicrobial peptides (AMPs) are valuable candidates for novel antimicrobial agents. AMPs clear bacterial infection through various mechanisms: disrupting the bacterial outer membrane, inducing reactive oxygen species (ROS) production, or interfering with bacterial metabolism.^[^ ^13^ ^]^ Due to the multiplexed mechanisms, AMPs are potentially advantageous against the MDR.^[^ ^14^, ^15^ ^]^ However, AMPs are metabolically unstable and exhibit potential toxic side effects such as high haemolytic activity hindering clinical development.^[^ ^16^, ^17^, ^18^, ^19^ ^]^

Multivalent strategies have long been proposed to improve binding efficiency, particularly in pathogen inhibition and cellular targeting.^[^ ^20^, ^21^, ^22^ ^]^ By simultaneously engaging multiple binding sites on the target, multivalent interactions significantly enhance the overall binding strength compared to monovalent approaches.^[^ ^23^, ^24^, ^25^ ^]^ This is especially critical when targeting pathogens like bacteria and viruses, which often have surface proteins or receptors with low binding affinities when engaged with monovalent ligands.^[^ ^26^, ^27^ ^]^ Our group's past work has shown that flexible multivalent 3D materials can effectively inhibit influenza A virus (IAV) infection and prevent its binding to host cells at low nanomolar concentrations.^[^ ^28^, ^29^ ^]^ Unlike viruses, which are dependent on host cells for their proliferation, bacteria, as independent unicellular organisms, can achieve high rates of self‐replication under suitable environmental conditions. It is therefore necessary to introduce a bactericidal agent into the multivalent binding system to inactivate bacteria and avoid the development of bacterial resistance. Based on these considerations, we hypothesized that integrating AMPs into a flexible multivalent 3D nanogel system could achieve specific eradication of bacteria while mitigating the toxicity concerns typically associated with free peptides.

Herein, we report a novel heteromultivalent nanogel (NG) system that is functionalized with Gal/Fuc ligands for the multivalent binding to LecA/LecB, and the cytotoxic BMAP‐18 peptide to avail a bactericidal effect against *P. aeruginosa*. (Figure 1a). Compared to previous reports, our design integrates lectin‐targeting sugars and antimicrobial peptides into a single nanogel platform, combining targeted adhesion with potent bactericidal activity. The heteromultivalent nanogel overcomes the limited bactericidal activity of monovalent sugar ligands and the instability/toxicity of free peptides, achieving eradication of *P. aeruginosa* in both planktonic and biofilm environments at a concentration of 8 µg mL^−1^, which is significantly better than BMAP peptide alone (16‐32 µg mL^−1^).^[^ ^30^, ^31^ ^]^

**Figure 1:**
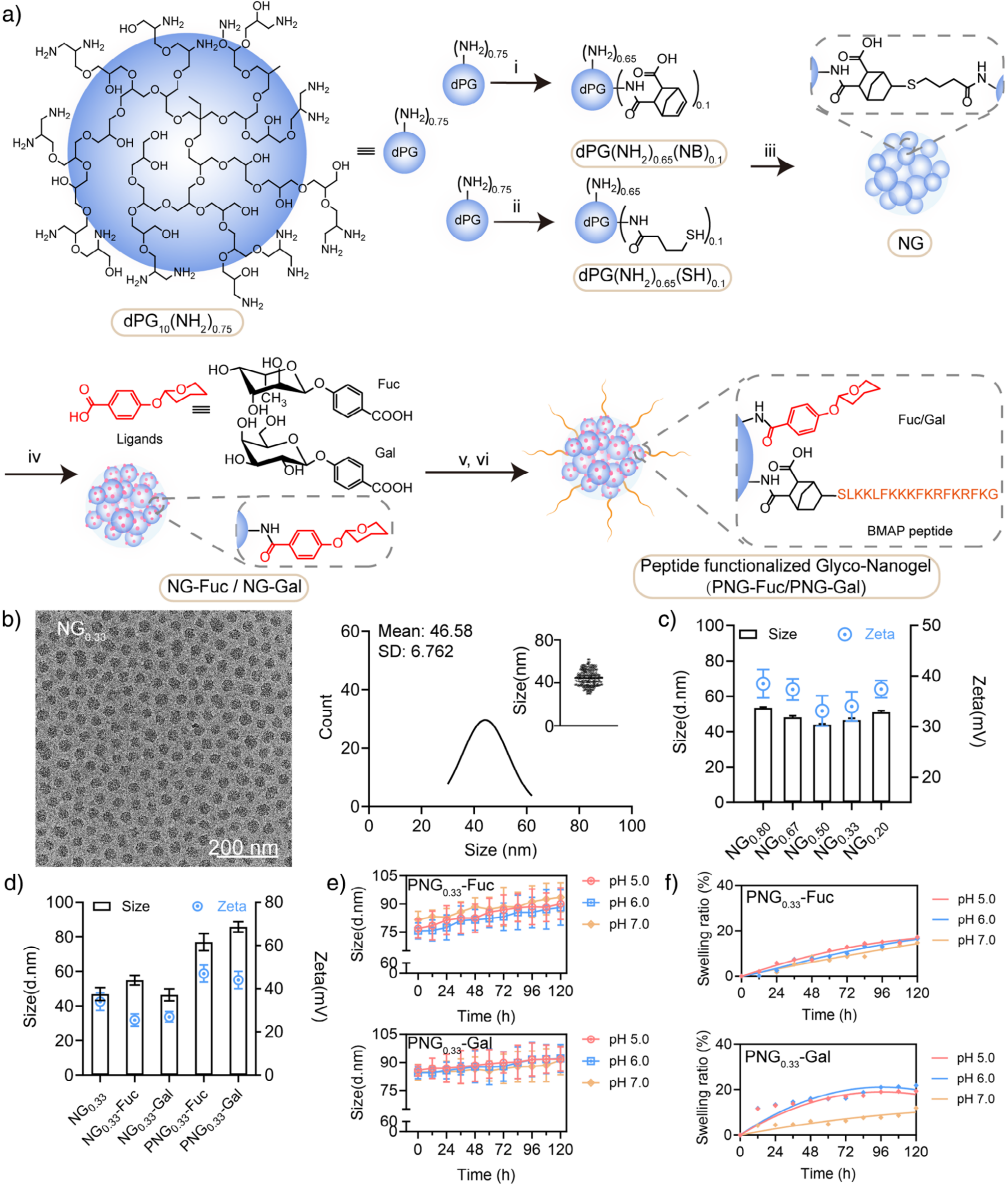
a) Schematics for the synthesis of nanogels (NGs). (i) Norbornene anhydride, DMF, room temperature, overnight. (ii), γ‐Thiobutyrolactone, DMF, room temperature, overnight. (iii), LAP, UV. (iv), NHS, EDC, deionized water, 8.0 pH, 24 h. (v), Norbornene anhydride, overnight. (vi), LAP, UV. b) Cryo‐EM Image for NGs and related size distribution by ImageJ software (scale bar = 200 nm), c) Size and Zeta potential for different NGs (mean ± SD, *n* = 3), d) Size and Zeta potential for different nanogels (mean ± SD, *n* = 3), e) Size for PNG‐Fuc/Gal with different pH environments (mean ± SD, *n* = 3), F) Swelling ratio for PNG‐Fuc/Gal with different pH environments compared with 0 h (mean ± SD, *n* = 3).

Nanogels can be constructed from diverse natural and synthetic polymers (e.g., polysaccharides, PEG, PNIPAM, polyglycerol) using methods such as emulsion polymerization, precipitation, self‐assembly, and click‐chemistry crosslinking.^[^ ^32^, ^33^ ^]^ Such compositional and methodological diversity provides opportunities for structural designing and the development of multifunctional biomedical platforms. For this work, nanogels (NGs) were prepared by photo‐induced thiol‐ene cross‐linking of 10 kDa dendritic polyglycerols (dPGs) with 10% norbornene group [dPG(NH_2_)_0.65_(NB)_0.1_] and 10 kDa dPG with 10% sulfhydryl group [dPG(NH_2_)_0.65_ (SH)_0.1_] (Figures S1.1–S1.5) using inverse nano precipitation technique.^[^ ^34^, ^35^ ^]^ Nanogels were obtained with low polydispersity index (PDI) and different degrees of crosslinking by changing the macromonomer ratio (Table 1).^[^ ^36^ ^]^ To assess the flexibility of NGs, we prepared hydrogels of the same formulation and measured their storage modulus. We observed increase in storage modulus from 0.4 kPa up to 1.5 kPa with increasing crosslinking between macromonomers (Figure S1.5). All NGs showed diameter around 50 nm and positive charge around +35 mV (Figure 1b,c). The positive zeta potential of NGs originates from the free NH_2_ on the nanogel surface.

**Table 1:** Composition, size, and zeta potential of different nanogels.

	Macromonomers						
NGs	dPG(NH_2_)_0.65_(NB)_0.1_ 20 wt% in water	dPG(NH_2_)_0.65_ (SH)_0.1_ 20 wt% in water	DI water (mL)	Acetone (mL)	Size by DLS (d ± SD) (nm)	PDI	ζ‐Potential (mV)
NG_0.80_	160	40	5	200	53.46 ± 0.53	0.15	+ 38.49 ± 2.79
NG_0.67_	134	66	5	200	48.18 ± 0.89	0.16	+ 33.37 ± 2.11
NG_0.50_	100	100	5	200	43.99 ± 0.51	0.15	+ 33.12 ± 3.00
NG_0.33_	66	134	5	200	46.66 ± 0.99	0.19	+ 34.00 ± 2.88
NG_0.20_	40	160	5	200	51.06 ± 0.80	0.18	+ 37.42 ± 1.71

^a)^ Suffix: dPG(NH_2_)_0.65_(NB)_0.1_ percentage of total starting material.

^b)^ In PBS (pH 7.4, 10 mM) at 1 mg mL^−1^.

^c)^ Polydispersity index obtained by DLS.

Next, we evaluated the binding potential of NGs with *P. aeruginosa* by a co‐culture of bacteria with Rhodamine‐labelled NGs. Despite the absence of any further modification, NG_0.33_ group showed the highest mean fluorescence intensity (MFI), (Figure S6) which indicated strongest binding that could be attributed to its optimal flexibility. Thus, NG_0.33_ was chosen as the substrate for the functionalization with fucose/galactose and antibacterial short‐chain peptides. DLS results showed that the fucose/galactose modification did not significantly affect the size or zeta potential, and the BMAP‐18 peptide modification increased both size and the zeta potential. The nanogels conjugated with both BMAP peptide and sugar (PNG_0.33_‐Fuc/PNG_0.33_‐Gal) were observed to have a size of approximately 80 nm and a zeta potential of 45 mV, compared to NG_0.33_, which had size of 47 nm and zeta potential of 35 mV (Figure 1d). Then, size of PNG_0.33_‐Fuc and PNG_0.33_‐Gal were measured in solutions with different pH 5.0–7.0 conditions, which closely resembles bacterial infection environment. No significant change in size was observed over five days incubation at different pH indicating the high stability of nanogels. (Figure 1e,f).

All functionalized nanogels were qualitatively assessed by flow cytometry to determine their binding ability to planktonic *P. aeruginosa*. As shown in Figure 2a,b, fucose/galactose conjugated nanogels, i.e., NG_0.33_‐Fuc and NG_0.33_‐Gal exhibited higher mean fluorescence intensity (MFI) than the control nanogel, i.e., NG_0.33_, respectively (Figure 2a,b), proving the important role of fucose/galactose for *P. aeruginosa* binding. Further conjugation of peptides did not improve the binding with *P. aeruginosa*. Next, mature *P. aeruginosa* biofilm was cultured to evaluate the binding of biofilm with nanogels (Figure 2). The NG_0.33_‐Fuc and NG_0.33_‐Gal exhibited substantially more co‐localized signals than the control and NG_0.33_ (Figure 2c,d). Additionally, nanogels conjugated with both BMAP‐18 peptide and Gal/Fuc ligands (PNG_0.33_‐Fuc and PNG_0.33_‐Gal) exhibited similar binding capacity as NG_0.33_‐Fuc and NG_0.33_‐Gal suggesting that the introduction of BMAP does not affect the binding of nanogels to *P. aeruginosa* (Figure 2).

**Figure 2:**
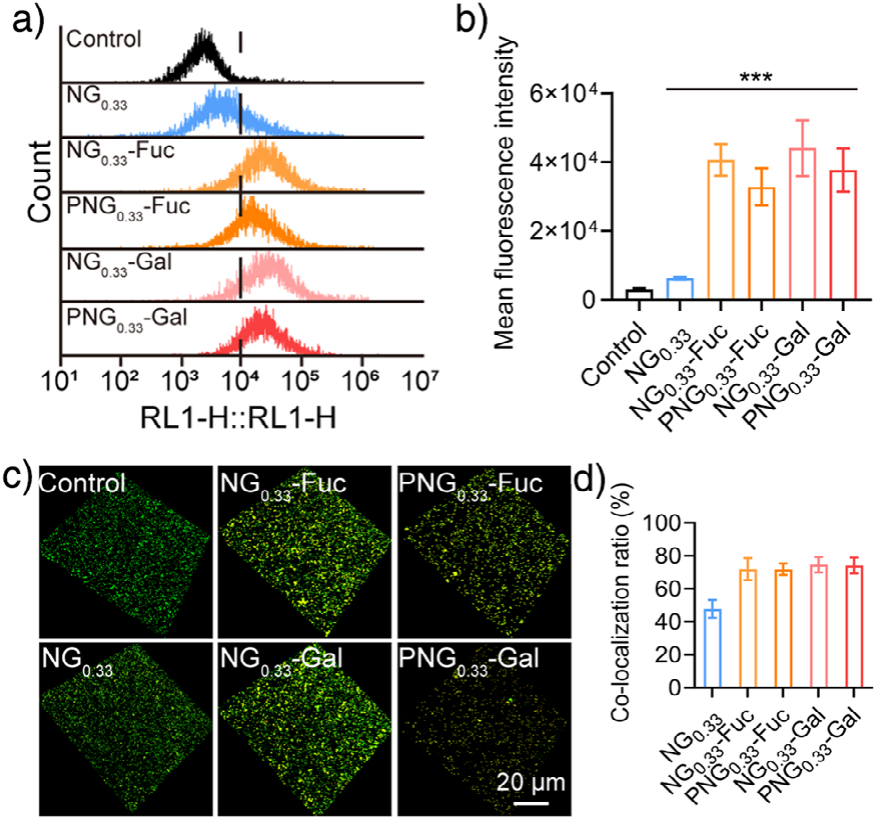
a) and b) Flow cytometry analysis of PNGs co‐incubated with planktonic *P. aeruginosa*., c) and d) *P. aeruginosa* biofilm binding capacity of different nanogels (green: FITC, P.a., Red: Rhodamine, yellow: co‐localization signal, Nanogels, scale bar = 20 µm), 0.01<*p< 0.1, 0.001<**p < 0.01, compared with the Control group.

Before evaluating the antibacterial activity of the nanogels, we also assessed their cellular toxicity (Figure S3–S5). Briefly, after a 24‐hour incubation with L929 cells, both PNG_0.33_‐Fuc and PNG_0.33_‐Gal maintained over 80% cell viability even at a high concentration of 1 mg/mL (Figure S5A,B). Additionally, no haemolysis was observed up to 1 mg mL^−1^ (Figure S5C,D), indicating that PNG_0.33_‐Fuc and PNG_0.33_‐Gal exhibit low toxicity towards normal somatic cells and erythrocytes. The bactericidal activity of nanogels against *P. aeruginosa* was then investigated by plate‐counting. In this measurement, NG_0.33_, BMAP alone, PNG_0.33_ (Gal/Fuc‐free), single sugar ligand, and NG_0.33_‐Fuc/NG_0.33_‐Gal treated groups were compared with PNG_0.33_‐Fuc/PNG_0.33_‐Gal group. After 12 h treatment with 8 µg mL^−1^ NG_0.33_‐Fuc/NG_0.33_‐Gal, the surviving bacteria were reduced to 10% of the control and single fucose/galactose groups (Figure 3a), which further supports the effectiveness of the multivalent binding strategy. However, bacterial “escape” from the multivalent inhibitors were observed from a 24 h growth curve monitor (Figure 3b). This suggests that mild ligand‐lectin binding is limited in clearing bacterial infections and the introduction of potent bactericidal components is needed. Compared to other groups, PNG_0.33_‐Fuc/PNG_0.33_‐Gal inhibited > 99.99% planktonic *P. aeruginosa* after 12 h treatment and displayed a continuous bactericidal efficacy for more than 72 h (Figure 3b,d). By Scanning Electron Microscopy (SEM), we observed severe morphological distortions and disintegration in the *P. aeruginosa* after 12 h treatment with PNG_0.33_‐Fuc/PNG_0.33_‐Gal (Figure 3c). Based on these results, introducing potent bactericidal components is as important as the sugar ligand functionalization. In our heteromultivalent system, the fucose/galactose ligand enabled initial inhibition of *P. aeruginosa* by efficient binding, while BMAP ensured a complete *P. aeruginosa* inactivation by membrane distortion.

**Figure 3:**
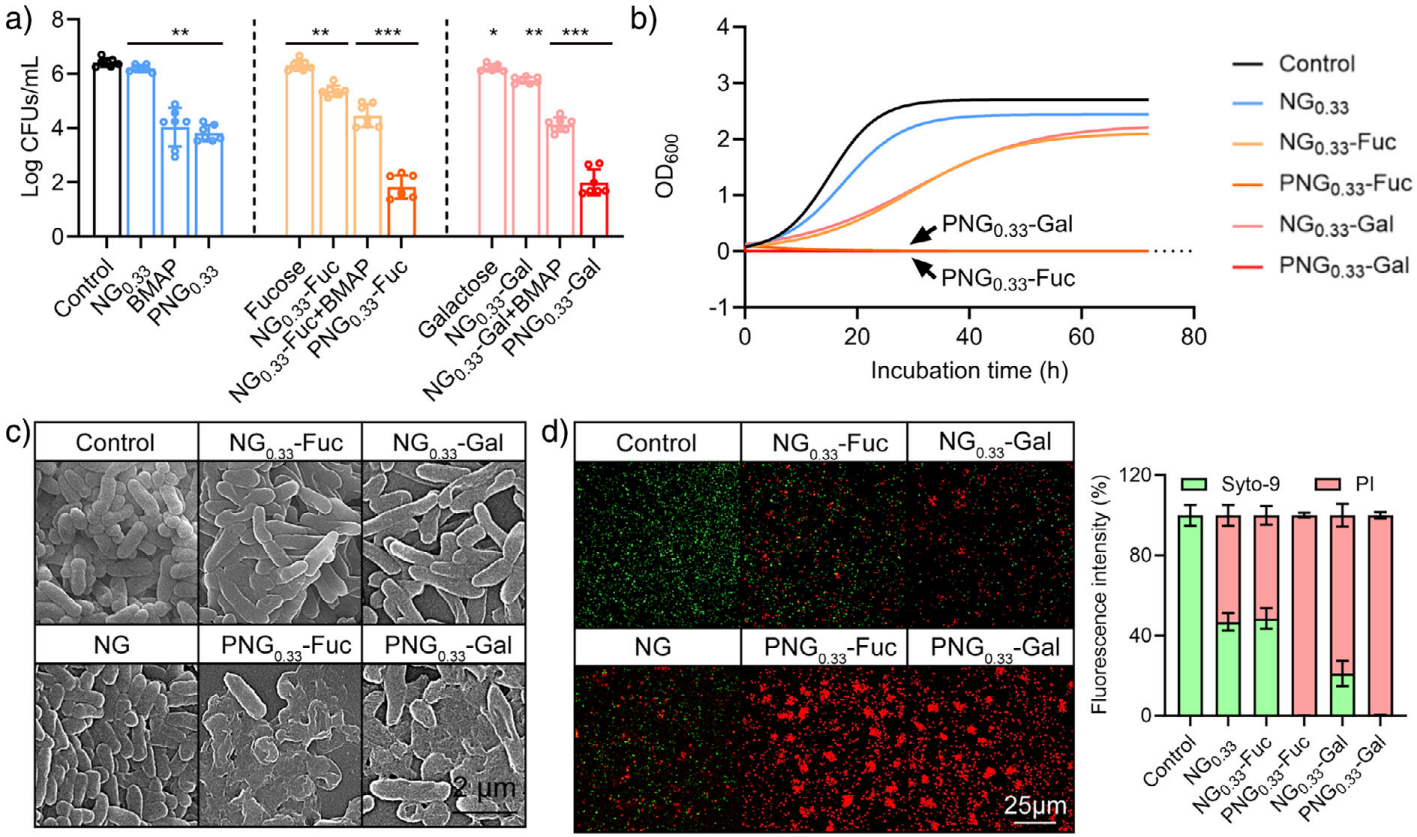
a) Planktonic *P. aeruginosa* survival ratio after 12 h of treatment with PNG0.33‐Fuc (mean ± SD, *n* = 7), b) 72 h growth curve after treatment with samples, c) SEM images for bacterial cells after different treatments (Black scale bar: 2 µm), d) Representative live/dead bacterial staining of *P. aeruginosa*. with different treatment and statistics for fluorescence intensity (green: live, red: dead, scale bar = 25 µm). 0.01<*p< 0.1, 0.001<**p < 0.01, ***p < 0.001, compare with Control group.

Inspired by the results of planktonic bactericidal experiments, we further evaluated the anti‐biofilm activity of the nanogels. After the 72 h co‐incubation with *P. aeruginosa*, compared to control and NG_0.33_, complete biofilm matrix removal was observed in the PNG_0.33_‐Fuc/PNG_0.33_‐Gal groups, while the glycosylated nanogels (NG_0.33_‐Gal/ NG_0.33_‐Fuc) or their physical mixtures with BMAP‐18 only slightly reduced biofilm matrix (Figure 4a and S9). This observation further reinforces our hypothesis that heteromultivalent nanogels with glyco‐residues and the BMAP‐18 peptide contribute to the long‐term eradication of *P. aeruginosa*. Further, a mature biofilm eradication assay was performed. After 12 h of treatment of 72 h mature biofilm, a 65% reduction in mean biofilm thickness and increased roughness were observed in the PNG_0.33_‐Fuc/PNG_0.33_‐Gal group, which was similar to the case of co‐incubation with tobramycin drug compared to the control (Figure 4c). More than 99.9% biofilm‐coated *P. aeruginosa* were inactivated (Figure 4a and S10). When the duration of treatment was extended to 24 h, the tobramycin did not show significant changes, while PNG_0.33_‐Fuc/PNG_0.33_‐Gal groups manifested 14% biofilm mean thickness and 3.5 times roughness compared to the control (Figure 4d), which means that the eradication of biofilm continued in the presence of PNG‐Fuc and PNG‐Gal. Overall, biofilm inhibition experiments with different control NG, NG‐Fuc/Gal and PNG‐Fuc/Gal reveal that PNG‐Fuc/‐Gal decomposes biofilm by a binding and killing mechanism. First, they bind with bacteria surface lectins via their specific sugar ligands and then inactivate using their surface BMAPs leading to a strong reduction in the biofilm matrix.

**Figure 4:**
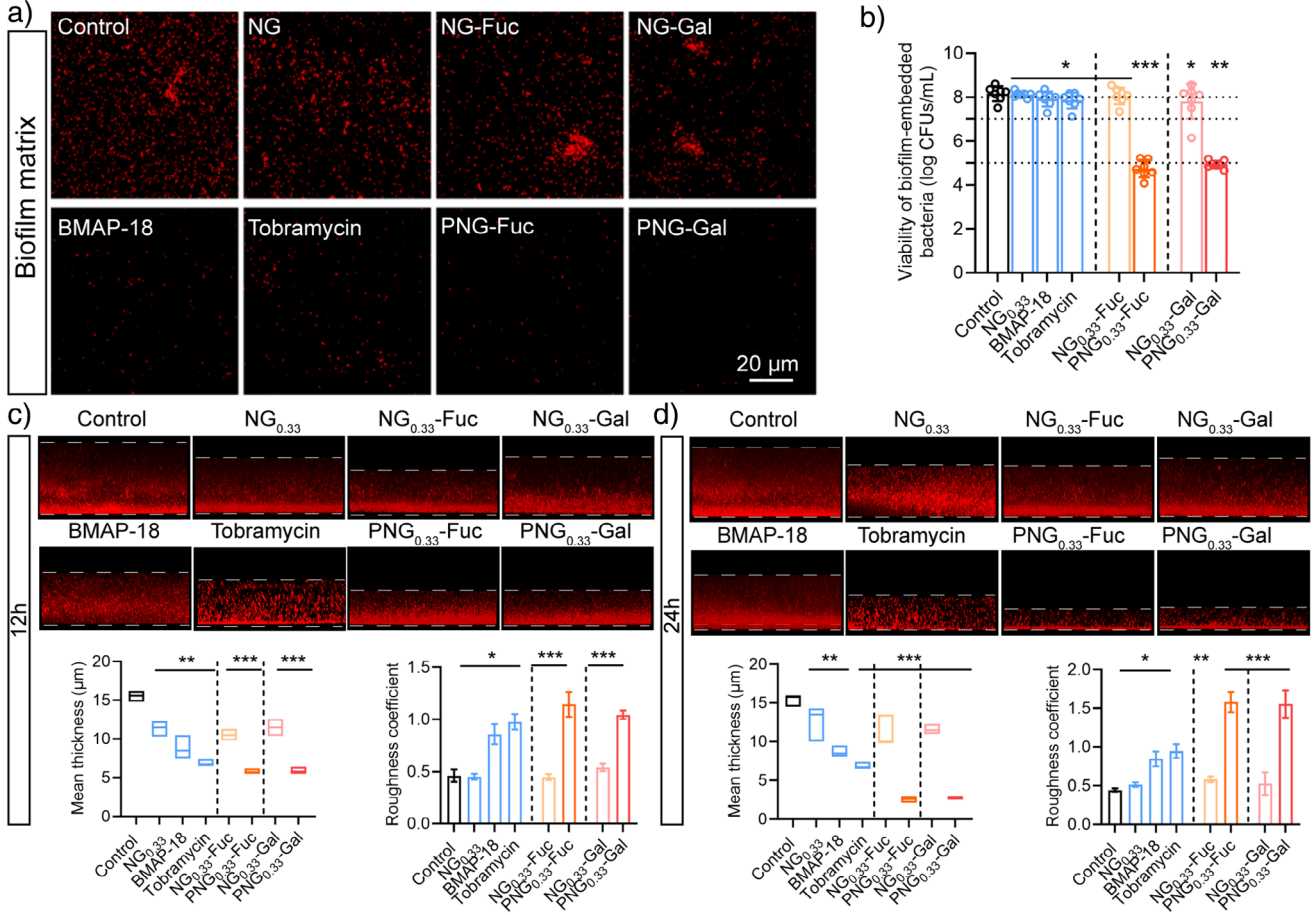
a) *P. aeruginosa* biofilm matrix visualization after 72 h of treatment with samples (red: biofilm matrix, scale bar = 20 µm), b) Biofilm coated‐ *P. aeruginosa* viability after 12 h treatment of samples. c) Mature *P. aeruginosa* biofilm visualization after 12 h of treatment with samples, and statistics for thickness and roughness (mean ± SD, *n* = 7), d) Mature *P. aeruginosa* biofilm visualization after 24 h of treatment with samples, and statistics for thickness and roughness (mean ± SD, *n* = 7). 0.01 <*p< 0.1, 0.001 <**p < 0.01, ***p < 0.001, compared with Control group.

Besides *P. aeruginosa*, *Escherichia coli* (*E. coli*) and *Methicillin‐resistant Staphylococcus aureus* (MRSA) were also used to evaluate the broad‐spectrum bactericidal potential of the nanogel systems (Figure S12). While PNG_0.33_‐Fuc and PNG_0.33_‐Gal were highly efficient against *P. aeruginosa* at 8 ug ml^−1^, a 90% killing of *E. coli* and MRSA were achieved at 32 and 16 µg mL^−1^, respectively (Figure S12A, B). This can be attributed to the naturally higher affinity that the galactose ligand shows for *E. coli* and MRSA lectins.^[^ ^37^, ^38^, ^39^ ^]^ Furthermore, the PNG_0.33_‐Fuc and PNG_0.33_‐Gal groups exhibited significantly lower biofilm biomass and biofilm‐encapsulated bacteria retention compared to the PNG_0.33_ group (Figure S12C‐F), consistent with the case for *P. aeruginosa* (Figure 4). We observe that while the disparity in the affinity with different bacterial lectins influences the bactericidal difference, the modification with BMAP still enables nanogel systems a broad‐spectrum antimicrobial potential.

In conclusion, we developed a novel heteromultivalent nanogel system for *P. aeruginosa* biofilm eradication. By integrating sugar ligands and BMAP‐18 peptide on the surface, the nanogel system combines specific bacterial adhesion with potent bactericidal activity, enabling strong multivalent binding to bacterial lectins while achieving sustained antibacterial efficacy for more than 72 h. PNG_0.33_‐Fuc/PNG_0.33_‐Gal achieved 99.99% inactivation of planktonic *P. aeruginosa* and 99.9% inactivation of biofilm‐coated *P. aeruginosa* within 12 h of treatment. Overall, this study demonstrates the broad‐spectrum antimicrobial potential of heteromultivalent nanogels, and highlights that such a modular design offers a promising avenue for the development of personalized and broad‐spectrum anti‐infective platforms.

## Supporting Information

The authors have cited additional references within the Supporting Information.^[^ ^40^, ^41^, ^42^, ^43^, ^44^, ^45^, ^46^, ^47^, ^48^, ^49^, ^50^, ^51^, ^52^, ^53^ ^]^ Supporting Information is available free of charge. Materials and methods, detailed synthetic protocols and reaction schemes, protocols for the biological assays, NMR figures, cytotoxicity analysis and related biological experiments protocols.

## Conflict of Interests

The authors declare no conflict of interest.

## Supporting information



Supporting Information

## Data Availability

The data that support the findings of this study are available in the Supporting Information of this article.
