# Supplementary material for: Heteromultivalent Nanogels as Highly Potent Inhibitors of Pseudomonas Aeruginosa
Source: Angew Chem Int Ed Engl. 2025 Nov 11;64(52):e13121. doi: 10.1002/anie.202513121 (PMC12723470; doi:10.1002/anie.202513121)
Supplement: Supplementary file 1 — Supporting Information [file ANIE-64-e13121-s001.docx]

Supporting Information

**Heteromultivalent Nanogels as Highly Potent Inhibitors of *Pseudomonas aeruginosa***

Yuhang Jiang^[a]^, Chuanxiong Nie^[a]^, Boyu Zheng ^[a]^, Vinod Khatri^[a]^, Denis Puccio ^[a]^, Yanping Long^[a]^, Mathias Dimde^[a]^, Rainer Haag*^[a]^, Sumati Bhatia*^[b]^

[a]	Y. Jiang, Dr. C. Nie, B. Zheng, V. Khatri, D. Puccio, Y. Long, Dr. M. Dimde, Prof. Dr. R. Haag,

Institute for Chemistry and Biochemistry, Freie Universität Berlin,

Takustraße 3, 14195 Berlin (Germany)

E-mail: haag@chemie.fu-berlin.de

[b]	Dr. S. Bhatia,

Department of Chemistry

Faculty of Science and Engineering, Swansea University,

Swansea, SA2 8PP, United Kingdom

sumati.bhatia@swansea.ac.uk

# Table of Contents

Table of Contents	1

1. Experiment Procedures	2

1.1 Materials	2

1.2 Synthesis	2

Synthesis of Fucose and Galactose derivatives	2

Synthesis of dPG(NH_2_)_0.65_(NB)_0.10_	4

Synthesis of dPG(NH_2_)_0.65_(SH)_0.10_	5

Synthesis of Nanogels	6

Dynamic Light Scattering (DLS)	7

Ellman's Assay	7

Cryo-Transmission Electron Microscopy (Cryo-TEM)	8

Macrorheology analysis	8

1.3 Biological assays	8

Cytotoxicity Assay	8

Haemolysis Assay	8

*Pseudomonas aeruginosa* (*P. a.*) Growth Inhibition Assay and Biofilm formation inhibition assay	9

Pseudomonas aeruginosa Growth Curve	9

*Pseudomonas aeruginosa* Biofilm Eradication Assay	9

*Pseudomonas aeruginosa* Biofilm Viability Assay	9

Scanning Electron Microscopy (SEM)	10

1.4 Statistical analysis	10

2. Results	10

3. References	19

# 1. Experiment Procedures

## 1.1 Materials

All solvents were of HPLC grade, and all chemicals were of at least 98% purity, unless otherwise noted. Sterilized PBS buffer (pH 7.4) was purchased from Gibco (Waltham, Massachusetts, the United States). All chemical solvents and reagents were obtained from commercial suppliers and used without further purification. For dry or oxygen-free reactions, the reaction is carried out under argon in Schlenk glassware. Merck silica gel 60 F254 pre-coated thin layer chromatography (TLC) plates are utilized for monitoring the progress of reactions in the case of small molecules. Staining in 5% H_2_SO_4_ in ethanol and ceric solution is used to visualize the spots on TLC. The purification of small molecules was done using a column having silica gel with a mesh size of 100-200. *Pseudomonas aeruginosa* (ATCC 10145), MRSA (ATCC 6538), human A549 and L929 cell lines were obtained from the American Type Culture Collection (ATCC), *E. coli ORN 178* was obtained from Max-Planck-Institut für Kolloid- und Grenzflächenforschung (MPIKG, Germany). 5-Norbornene-2,3-dicarboxylic anhydride and 4-Butyrothiolactone (97%) were purchased from ABCR (Karlsruhe, Germany). BMAP-18 peptide (GRFKRFRKKFKKLFKKLS, 97%) was obtained from NovoPro (Shanghai, China). Unless stated otherwise, all other chemicals were obtained from Merck (Darmstadt, Germany). The dialysis tubing used in this work was made of regenerated cellulose (RC), and the dialysate was replaced every 4 - 6 hours during the dialysis process. Ultrafiltration centrifuge tubes (Amicon® Ultra Zentrifugenfilter) were purchased from Sigma Aldrich (Darmstadt, Germany), and the membrane material was PES or RC.

NMRs were recorded on Bruker AMX 700 (700 MHz) or Bruker AMX 400 (400 MHz) spectrometer and calibrated for peaks of deuterated solvents. Fourier transform infrared (FTIR) spectroscopy were recorded transmittance (%) at wavenumbers from 450 to 4000 cm^-1^ on a Fourier Transform Infrared spectrometer.

The amino and thiol content were detected by TNBS colorimetry and Ellman's test, respectively. For nanogels, the accessible amino groups were quantified by TNBS assay and are expressed as μmol –NH_2_/mg nanogel. The sugar ligand content was quantified by ultraviolet spectroscopy (UV spectroscopy) using a full-spectrum microplate reader (Tecan, the United States). A standard curve (5 – 100 μM) was established with λ_max_ of 260 nm. The sample was blanked with NGs to subtract the scattering, and the concentration was calculated according to the Beer–Lambert law and expressed as μmol ligand/mg nanogel.

## 1.2 Synthesis

The dendritic polyglycerol amine (dPG, M_n_=8.5 kDa, Mw = 12.6 kDa) used for the experiments were synthesized according to the procedure already reported and their molecular weights were verified by GPC (H_2_O + 0.3 M formic acid, 3 mg/mL, 1 mL/min) coupled with refractive index detector (Agilent, Germany)^[1]^. The amino content was measured by TNBS colorimetry and was 75.42 %.

^1^H NMR (700 MHz, DMSO-d_6_) δ = 4.00 - 3.27 (m, dPG backbone) ppm.

^13^C NMR (176 MHz, DMSO-d_6_) δ = 76.25, 72.45, 69.88, 62.28, 46.88, 43.15 ppm.

HSQC (700 MHz, ^1^H; 176 MHz, ^13^C, DMSO-d_6_) δ = H 3.0 – 4.0 & C 60 – 80 (dPG backbone), H 2.7 – 3.3 & C 46 – 43 (dPG) ppm.

HMBC (700 MHz, ^1^H; 176 MHz, ^13^C, DMSO-d_6_) δ = H 3.0 – 4.0 & C 60 – 80 (dPG backbone), H 2.7 – 3.3 & C 46 – 43 (dPG) ppm.

### Synthesis of Fucose and Galactose derivatives

**Synthesis of 4-(*α*-L-fucopyranosyloxy)-benzoic acid**

**Scheme 1**. Synthesis of 4-(*α*-L-fucopyranosyloxy)-benzoic acid.

The peracetylated L-fucose was synthesized as per the literature procedure and obtained in 97% yield. This compound is identified on the basis of their spectral data (^1^H, ^13^C NMR spectra and HRMS) analysis. The structure was further confirmed by comparison of its physical and spectral data with those reported in the literature.^[2]^

**Benzyl-4-(2',3',4'-tri-*O*-acetyl-*α*-L-fucopyranosyloxy)-benzoate**

To a solution of compound (5.5 g, 16.56 mmol, 1 equiv) in DCM (70 mL) was added benzyl-4-hydroxybenzoate (7.5 g, 33.12 mmol, 2 equiv). The resulting reaction mixture was cooled to 0 ^o^C and BF_3_.EtO (6.1 mL, 49.68 mmol, 3 equiv) was added. The reaction mixture was warmed to room temperature and allows stirring overnight. After neutralizing the reaction mixture with saturated solution of NaHCO_3_, DCM (2 x 50 mL) was used to extract the compound. The combined organic layer was dried over Na_2_SO_4_, filtered and then concentrated at reduced pressure to give the crude product, which was purified over a silica gel column using 20% ethyl acetate in petroleum ether as eluent to afford the pure product. It was obtained as white solid (7.2 g) in 87 % yield. M.pt. 126-128 ^o^C.

^1^H NMR (600 MHz, CDCl_3_) *δ* = 8.03 (d, *J*=8.8, 2H), 7.42 (d, *J*=7.2, 2H), 7.37 (t, *J*=7.3, 2H), 7.33 (t, *J*=7.2, 1H), 7.08 (d, *J*=8.8, 2H), 5.81 (d, *J*=3.6, 1H), 5.56 (dd, *J*=10.9, 3.3, 1H), 5.35 (d, *J*=2.9, 1H), 5.33 (s, 2H), 5.28 (dd, *J*=10.9, 3.6 1H), 4.20 (q, *J*=6.4, 1H), 2.18 (s, 3H), 2.04 (s, 3H), 2.02 (s, 3H), 1.10 (d, *J*=6.5, 3H) ppm.

^13^C NMR (151 MHz, CDCl_3_) *δ* = 170.6, 170.2, 165.9, 160.2, 136.2, 131.8, 128.6, 128.3, 128.2, 124.5, 116.0, 94.5, 70.8, 67.9, 67.6, 66.6, 65.8, 20.8 (2 peaks), 20.7, 15.9 ppm.

HRMS (ESI) m/z: [M+Na]^+^ calcd for C_26_H_28_NaO_10_ 523.1575, Found 523.1584.

IR (neat): 1748, 1728, 1608, 1515, 1376, 1364, 1226, 1217, 1179, 1084, 1051, 974, 914, 855, 771, 730, 698 and 685 cm^-1^.

**4-(*α*-L-Fucopyranosyloxy)-benzoic acid**

To a solution of compound (12 g, 24 mmol, 1 equiv) in THF (120 mL) was hydrogenated over Pd/C (1.2 g, 10% of VK-018, ~10 wt% of substrate) under H_2_ atmosphere at room temperature for 2h. The reaction mixture was filtered through a celite pad, washed with THF (50 mL) and the solvent was removed under reduced pressure to afford the white solid compound (9.2 g). The crude product thus obtained was further hydrolysed using 2M NaOH solution (50 mL). The reaction mixture was stirred for 2h followed by neutralization with 1M HCl solution. The resulting reaction mixture was concentrated under reduced pressure to obtain the crude product, which was passed over a silica gel column using 20% MeOH in CHCl_3_ as eluent to afford the pure product.

It was obtained as white amorphous solid (5.2 g) in 81.6 % yield.

^1^H NMR (600 MHz, CDCl_3_) *δ* = 7.97 (d, *J*=8.9, 2H), 7.19 (d, *J*=8.9, 2H), 5.74 (d, *J*=3.8, 1H), 4.13-4.09 (m, 2H), 3.98 (dd, *J*=10.3, 3.8, 1H), 3.86 (d, *J*=3.2, 1H), 1.14 (d, *J*=6.6, 3H) ppm.

^13^C NMR (151 MHz, CDCl_3_) *δ* = 170.5, 159.2, 131.4, 128.1, 116.4, 96.7, 71.7, 69.5, 67.8 (2 peaks), 15.3 ppm.

HRMS (ESI) m/z: [M+Na]^+^ calcd for C_13_H_16_NaO_7_ 307.0789, Found 307.0799.

IR (neat): 3348-2529, 1690, 1609, 1587, 1513, 1427, 1314, 1239, 1169, 1080, 1039, 1020, 966, 853, 777, 746 and 683 cm^-1^.

**4-(*β*-D-Galactopyranosyloxy)-benzoic acid**

**Scheme 2**. Synthesis of 4-(*β*-D-galactopyranosyloxy)-benzoic acid.

Compound Gal-Acid was synthesized from peracetylated D-galactose in two steps as per the literature procedure.^[3]^ The crude product (2 g, 1 equiv) thus obtained was further hydrolysed using 2M NaOH solution (20 mL). The reaction mixture was stirred for 3h followed by neutralization with 1M HCl solution. The resulting reaction mixture was concentrated under reduced pressure to obtain the crude product, which was passed over a silica gel column using 30 % MeOH in CHCl_3_ as eluent to afford the pure product. It was obtained as white solid (1.1 g) in 86% yield. M.pt. 191-193 ^o^C.

This compound is identified on the basis of their spectral data (^1^H, ^13^C NMR spectra and HRMS) analysis. The structure was further confirmed by comparison of its physical and spectral data with those reported in the literature.^[4]^

### Synthesis of dPG(NH_2_)_0.65_(NB)_0.10_

The norbornane-functionalized globular dendritic polyglycerol (dPG(NH_2_)_0.65_(NB)_0.1_) was prepared by dissolving dPG(NH_2_)_0.75_ (10 mg, 1 equiv) and norbornane anhydride (2.35 mg, 14 μmol, 14 equiv) in DMF (20 mL) stirred overnight at room temperature (200 rpm). Then DMF was evaporated (24 ℃), and the residue was dialyzed against deionized water using a dialysis membrane with MWCO = 1 kDa to obtain an aqueous solution of dPG(NH_2_)_0.65_(NB)_0.10_. The solvent was further reduced by rotary evaporation to adjust the solution concentration to 50 mg/mL and stored at 4°C until use. Structures were observed by ^1^H NMR, ^13^C NMR, HSQC and HMBC (Bruker AMX 700, 700 MHz) and FTIR (PerkinElmer, Spectrum Two). The amino content was measured by TNBS colorimetry and was 63.57 %.

^1^H NMR (700 MHz, DMSO-d_6_) δ = 6.06, 6.03 (d, 2 NB-H), 4.00-2.92 (m, dPG backbone, NB-H, H_2_O), 1.55 (m, 2 NB-H) ppm.

^13^C NMR (176 MHz, DMSO-d_6_) δ = 177.83, 145.85, 134.75, 78.12, 72.81, 71.15, 52.14, 45.66, 44.55 ppm.

HSQC (700 MHz, ^1^H; 176 MHz, ^13^C, DMSO-d_6_) δ = H 3.0 – 4.0 & C 60 – 80 (dPG backbone, NB), H 2.9 – 3.3 & C 44 – 53 (NB), H 6.06 – 6.03 & C 177.83 (NB), H 1.55 & C 52.14 (NB) ppm.

HMBC (700 MHz, ^1^H; 176 MHz, ^13^C, DMSO-d_6_) δ = H 3.0 – 4.0 & C 60 – 80 (dPG backbone, NB), H 6.06 – 6.03 & C 44-53 (NB ppm).

IR (cm^-1^) ν: 3357, 2914, 2866, 2030, 1691, 1395, 1077, 1027, 845, 774, 728, 663, 614, 553, 491 ppm.

**Scheme 3**. Synthesis of dPG(NH_2_)_0.65_(NB)_0.10_.

### Synthesis of dPG(NH_2_)_0.65_(SH)_0.10_

A one-step ring opening reaction were used to prepared the thiol-based functionalized globular dendritic polyglycerol (dPG(NH_2_)_0.65_(SH)_0.10_), briefly, dPG(NH_2_)_0.75_ (10 mg, 1.0 μmol, 1 equiv) and γ-Thiobutyrolactone (1.33 mg. 13 μmol, 13 equiv) were dissolved in DMF (25 mL).^[5-7]^ The solution was stirred overnight at room temperature with the argon environment , then DMF solvent was evaporated (24 ℃) and purification by ultrafiltration (5000 g, 15 min X 3, MWCO = 5 kDa) with deionized water, the resulting fresh product was immediately used in next experiments or stored at -20°C. Structure were observed by ^1^H NMR, ^13^C NMR, HSQC and HMBC (Bruker AMX 700, 700 MHz) and FTIR (PerkinElmer, Spectrum Two), the degree of sulfhydryl modification was determined by Ellman's test (10.55 %). The amino content was measured by TNBS colorimetry and was 64.12 %.

^1^H NMR (700 MHz, D_2_O) δ 4.00-3.25 (m, dPG backbone), 2.90-2.65 (m, 2H), 2.40-2.20 (m, 2H), 1.95-1.85 (m, 2H) ppm.

^13^C NMR (176 MHz, DMSO-d_6_) δ = 175.27, 76.26, 75.14, 73.55, 62.38, 35.56, 34.24, 30.75, 26.88 ppm.

HSQC (700 MHz, ^1^H; 176 MHz, ^13^C, DMSO-d_6_) δ = H 3.3 – 4.0 & C 60 – 80 (dPG backbone), H 1.9 – 2.8 & C 25 - 36 (–CONH–(CH_2_)_3_-) ppm.

HMBC (700 MHz, ^1^H; 176 MHz, ^13^C, DMSO-d_6_) δ = H 3.3 – 4.0 & C 60 – 80 (dPG backbone), H 1.9 – 2.8 & C 25 - 36 (–(CH_2_)_3_-SH), H 1.9 – 2.8 & C 173 – 178 (–CONH-) ppm.

IR (cm^-1^)ν: 3417, 2931, 2867, 2585, 1726, 1658, 1497, 1439, 1410, 1388, 1255, 1094.

**Scheme 4**. Synthesis of dPG(NH_2_)_0.65_ (SH)_0.1_.

### Synthesis of Nanogels

For the preparation of NG_0.33_, dPG(NH_2_)_0.65_(NB)_0.10_ (6.6 mg, 0.66 μmol, 1 equiv) and freshly prepared dPG(NH_2_)_0.65_(SH)_0.10_ (13.4 mg, 1.34 μmol, 2 equiv) were added in Milli-Q water (5 mL), solutions were then added to the stirred acetone (200 mL) at 250 rpm. Then lithium phenyl-2,4,6-trimethylbenzoylphosphinate (LAP, 50 μg, 250 nmol, 0.4 equiv) were quickly added in system and irradiated at 360 nm using the UV lamp (10 mW/cm², 366 nm, Merck) for 15 min. The reaction was stopped immediately after the initiation and the dispersion was allowed to stand for 12 h in the dark environment. Then acetone was evaporated to obtain nanogel dispersions in water, and the dialysis method was used for the purification. The concentration of the nanogel was determined by lyophilizing a small portion of the solution and weighing it. The nanogel stock solution was stored at 4℃, structurally characterized by FTIR (PerkinElmer, Spectrum Two), meanwhile stored the nanogel to 4℃ until use. The rest of NGs were prepared following the same process as for the preparation of NG_0.33_ (**Table 1**). The amino content was measured by TNBS colorimetry (μmol -NH_2_/mg): NG_0.80_ 2.65, NG_0.67_ 2.14, NG_0.50_ 1.85, NG_0.33_ 2.23, NG_0.20_ 2.79.

IR (cm^-1^) ν: 3423, 2931, 1655, 1497, 1438, 1409, 1386, 1255, 1093, 1031, 865.

**Scheme 5**. Synthesis of Nanogel (NG).

**Scheme 6**. Synthesis of NG-Fuc/NG-Gal.

**Post Functionalization of Nanogels.**

The carboxylated galactose/fucose ligand (5.40 mg, 18 μmol, 1 equiv), were dissolved in deionized water (10 mg/mL), and the pH was adjusted to 8.0 by adding NaOH solution dropwise. Then, added (2.49 mg, 21.6 μmol, 1.2 equiv), and EDC (4.14 mg, 21.6 μmol, 1.2 equiv) and stirred for one hour to obtain the active solution. The active solution was then slowly dripped into the nanogel solution in deionized water (5 mg conc. 1 mg/mL, 4.3 equiv.) and stirred for 24 hours to produce fucose/galactose functionalized nanogel (NG-Fuc/NG-Gal). The resulting nanogel solution was dialyzed through deionized water for purification (72 h, MWCO=5 kDa), the concentration was determined by lyophilizing a small portion of the aqueous nanogel solution to determine the concentration and stored the nanogel to 4°C until use. Structure was observed by FTIR (PerkinElmer, Spectrum Two). The amino content was measured by TNBS colorimetry (μmol -NH_2_/mg): NG_0.33_-Fuc 1.52, NG_0.33_-Gal 1.48.

Ligand content (μmol ligand/mg nanogel.): NG_0.33_-Fuc 0.71, NG_0.33_-Gal 0.75.

NG_0.33_-Fuc: IR (cm^-1^) ν: 3432, 3218, 2652, 1672, 1545, 1488, 1404, 1385, 1232, 1096, 1068.

NG_0.33_-Gal: IR (cm^-1^) ν: 3428, 3220, 2598, 1702, 1533, 1500, 1402, 1365, 1212, 1085, 1058.

For the preparation of D-BMAP18 functionalized Nanogel PNG-Fuc/PNG-Gal, Norbornane anhydride (9.85 mg, 6 μmol, 1 equiv) was added to the NG-Fuc/NG-Gal solution (5 mg, 1 mg/mL) and stirred overnight, then D-BMAP18 (4 mg, 1.79 μmol, 0.3 equiv), and LAP (5 μg, 25 nmol, 0.004 equiv) were added, and reaction was irradiated at 360 nm by the UV lamp (10 mW/cm², 366 nm, Merck) for 15 min. After initiation the dispersion was allowed to stand for 12 h in the dark environment. Then purified by dialysis with deionized water (72 h, MWCO=10 kDa), and the solvent was reduced by rotary evaporation (40°C) to obtain a nanogel dispersion with a concentration of 20 mg/mL. Size and zeta potential were observed by DLS. Structure was observed by FTIR (PerkinElmer, Spectrum Two). The amino content was measured by TNBS colorimetry (μmol -NH_2_/mg): PNG_0.33_-Fuc 1.21, PNG_0.33_-Gal 1.19.

PNG_0.33_-Fuc: IR (cm^-1^) ν: 3425, 3223, 2980, 1655, 1485, 1465, 1398, 1363, 1244, 1105, 1051, 855, 742. PNG_0.33_-Gal: IR (cm^-1^) ν: 3418, 3195, 2952, 2600, 1655, 1492, 1465, 1395, 1366, 1288, 1098, 1046, 846, 758.

**Scheme 7**. Synthesis of PNG-Fuc/PNG-Gal.

### Dynamic Light Scattering (DLS)

The particle size distribution and ζ-potential of the nanogels were measured by a Zetasizer Ultra (Malvern, Cambridge, United Kingdom), where the nanogels were dispersed in phosphate buffered saline (PBS) at a concentration of 1 mg/mL and equilibrated for 60s in the Zetasizer Ultra before measurement.

### Ellman's Assay

Ellman’s reagent 5,5'-Dithio-bis (2-nitrobenzoic Acid) (DTBN) was dissolved in PBS buffer to obtain test solution (5 mM) and stored at 4°C in the dark environment until use. L-cysteine was dissolved in PBS buffer (1 mM) to obtain standard solution, which was diluted to 0, 5, 10, 20, 40, 60, 80, 100 μM for standard curve determination. 50 μL diluted standard solution was mixed with 150 μL test solution, and then placed at room temperature for 15 min, the absorbance at 412 nm was measured by plate reader (Tecan, the United States) and the standard curve was fitted by A linear regression mode. The same procedure was followed to measure the absorbance at 412 nm for dPG(NH_2_)_0.65_(SH)_0.10_ and NGs to determine sulfhydryl content.

### Cryo-Transmission Electron Microscopy (Cryo-TEM)

The microscopic morphology of the nanogels was visualized by cryo-electron microscopy (Cryo-EM), perforated carbon film-covered microscopical 200 mesh grids (R1/4 batch of Quantifoil, MicroTools GmbH, Jena, Germany) were cleaned with chloroform and hydrophilized by 60 s glow discharging at 10 mA in a Safematic CCU-010 device (safematic GmbH, Zizers, Switzerland). Subsequently, 4 μL aliquots of the sample solution were applied to the grids. The samples were vitrified by automatic blotting and plunge freezing with a FEI Vitrobot Mark IV (Thermo Fisher Scientific Inc., Waltham, Massachusetts, USA) using liquid ethane as cryogen. The vitrified specimens were transferred to the autoloader of a FEI TITAN KRIOS G3i TEM. This microscope is equipped with a high-brightness field-emission gun (XFEG) operated at an acceleration voltage of 300 kV. Micrographs were acquired on a Falcon3EC detector (Thermo Fisher Scientific Inc., Waltham, Massachusetts, USA). Images were conducted with a nominal magnification of 22,500x corresponding to a pixel size of 3.70 Å and a defocus of -4 µm. The total electron flux applied was set to 42 el/Å2 accumulated over an exposure time of 3.21 s.

### Macrorheology analysis

For Macrorheology, 20% wt of aqueous solutions of dPG(NH_2_)_0.65_(NB)_0.1_ and dPG(NH_2_)_0.65_ (SH)_0.1_ were used to prepare hydrogels with the same precursor ratio of nanogels. The storage modulus of the hydrogel was then extracted by the Kinexus rheometer (NETZSCH GmbH, Selb, Germany) on a conical plate with a diameter of 20 mm (25 °C, mean normal force maintained at ≈0.1 N, 1% strain, frequency 0.1-10 Hz).

## 1.3 Biological assays

### Cytotoxicity Assay

Cytotoxicity was tested using the live/dead staining method and the CCK-8 test. For cytotoxicity assay, L929 cells (4×10^3^ cells/well) were seeded in a 96-well plate, and 100 μL DMEM medium (10% FBS, 1% P/S) was added to each well. After 24 h incubation under 37℃ and 5% CO_2_ environment, samples (NGs, NG-Fuc/NG-Gal, PNG-Fuc/PNG-Gal) were added into wells with various concentrations (1 μg/mL, 10 μg/mL, 100 μg/mL, and 1000 μg/mL) and cultured for another 24 hours. Then Calcein AM and propidium iodide were used to stain live and dead cells, respectively. The fluorescence microscope (Zeiss) was used for visualization.

For CCK-8 assay, samples (NGs, NG-Fuc/NG-Gal, PNG-Fuc/PNG-Gal) were co-incubated with L929 cells with planned concentration (1 μg/mL, 10 μg/mL, 100 μg/mL and 1000 μg/mL) for 24 hours, then used CCK-8 treated cells and measured the 450 nm absorbance by plate reader (Tecan, the United States), no sample group was blank control.

### Haemolysis Assay

For fresh human blood, serum was removed by centrifugation (2500 rpm, 10 min), then washed with PBS three times to collect red blood cells (RBCs), and RBCs were diluted with PBS to get the 4% RBCs suspension. Then 900 μL (1 mg/mL) samples and 100 μL RBCs suspension were added to the EP tube and incubated for 1 h at 37℃. After co-incubation, the supernatant was collected by centrifuge (6000 rpm, 5min), and the absorbance of the supernatant at 545 nm was tested by a microplate reader (Tecan, the United States). Setting Triton-X 100 as positive control and PBS as negative control.

### *Pseudomonas aeruginosa* (*P. a.*) Growth Inhibition Assay and Biofilm formation inhibition assay

Biofilm was quantified by crystal violet ^[8-9]^ and visualized by Filmtracker Red^[10-11]^. First, *P.a.* were seeded on the LB agar and cultured overnight with 37 ℃, then a single colony was added in 5 mL LB Medium in bacterial culture falcon. After 6 hours of culture, *P.a.* (10^5^ CFUs/mL) were seeded in 96-well plates and samples (Fuc/Gal ligand, BMAP18, NGs, NG-Fuc/NG-Gal, PNG-Fuc/PNG-Gal) were added in wells at planned concentrations. After co-incubation for an additional 24 hours, the OD_600_ was measured by a plate reader (Epoch2).

For the Biofilm inhibition assay, samples at planned concentration and *P.a.* (10^5^ CFUs/mL) were co-incubated in 48-well plates for 72 hours, after incubation, the wells were washed with PBS. Then, 0.1% crystal violet was added and incubated with the biofilm for 10 mins to stain biofilm matrix. After staining, excess of crystalline violet solution was removed by washing with Miliq water (10 min, 3 times), the stained biofilm was dissolved by ethanol, and a plate reader (Epoch2) measured biofilm biomass (Absorbance at 595 nm). Filmtracker Red (Thermofisher) was used to visualize the biofilm matrix, and CLSM (Leica, Germany) was used to capture images.

### Pseudomonas aeruginosa Growth Curve

For growth curve measurement, a single colony *P.a.* was taken in 5 mL LB medium and cultured for 6 hours, then co-incubated with samples and *P.a.* (10^5^ CFUs/mL) in 48-well plates for 72 hours, and a plate reader (Epoch2) was used to read the OD_600_ of all wells every 30 mins. The growth curve fitting function of the software Origin 2023 was used to fit growth curves.

### *Pseudomonas aeruginosa* Biofilm Eradication Assay

*P.a.* was cultured in 5 mL of LB medium for 6 hours to get the bacterial solution. Then, they were seeded in 48-well plates (10^5^ CFUs/mL) for 72 hours. Then, sterilized PBS buffer was used to wash each well three times to obtain mature biofilm. For mature biofilm, they were co-incubated with samples for 24 hours, all liquids were removed, and all wells were washed with sterilized PBS buffer, then added 0.1% crystal violet to stain biofilm (200 μL/well, incubate 20min). Excess crystalline violet solution was aspirated and washed with Miliq water (10 min,3 times), followed by ethanol to dissolve the stained biofilm. The plate reader (Epoch2) was used to read the absorbance at 595 nm of all wells to get biofilm biomass. Filmtracer Red (Thermofisher) was used to visualize the biofilm matrix, and CLSM (Leica, Germany) was used to capture images.

### *Pseudomonas aeruginosa* Biofilm Viability Assay

Biofilm viability was examined using confocal microscopy (Leica, Germany) and the WST method.^[12-13]^ For the confocal microscopy measurement, *P.a.* was seeded in 8-well confocal plate (1× 10^8^ CFUs/mL, 200 μL/well) and cultured for 72 hours in incubator under 37℃ to get the mature biofilm. After incubation, nanogel suspensions were added in each well and incubated for 12 hours. Then, bacterial live-dead kits (ThermoFisher, the United States) were used to distinguish between live and dead bacteria. A confocal microscope (Leica, Germany) was used to get fluorescent images.

For survival rate, homogeneous suspension of bacteria was obtained by vortexing to disrupt the structure of the mature biofilm, and subsequently 20 μL drops of the suspension were taken and incubated on LB Agar overnight and counted for CFUs.

For WST assay, mature biofilms were obtained by the same method as in the confocal microscopy measurement, and then biofilm viability kits (Dojindo, Japan) were used to test the biofilm viability.

### Scanning Electron Microscopy (SEM)

P. a. was seeded on LB agar and cultured overnight in incubator at 37°C. Then, a single colony was inoculated into 5 mL of LB medium in a bacterial culture Falcon tube. After 6 hours of incubation, NG_0.33_, NG_0.33_-Fuc, NG_0.33_-Gal, PNG_0.33_-Fuc, and PNG_0.33_-Gal were added separately to the culture Falcon tubes and co-incubated with P. aeruginosa for another 2 hours. At the end of the incubation, the medium was removed by centrifugation (1000 rpm, 5 min) and the precipitate was collected, followed by the addition of 5 mL of PBS buffer to wash the precipitate three times and centrifugation again to collect the precipitate. Collections were fixed using 4% paraformaldehyde for 0.5 h and dehydrated progressively with different concentrations of ethanol (30%, 50%, 70%, 90%, 100%, 100%, 10 mins each concentration). For dehydrated products, Sputter instrument Safematic CCU-010 LV (safematic GmbH, Zizers, Switzerland) was used to metal coat the sample to enhance the electronic signal. And then SEM Hitachi SU8030 (Hitachi High-Tech Corporation, Japan) was used to capture images.

## 1.4 Statistical analysis

All data were expressed as mean ± SD from at least triplicated experiments. Differences between the two groups were analyzed by a Two-way ANOVA. P < 0.1 were considered statistically significant.

## 2. Results

**Figure S1.1**. (A) ^1^H NMR (DMSO-d_6_, 700 MHz) spectrum of dPG(NH_2_)_0.75_, dPG(NH_2_)_0.65_(NB)_0.1_ and dPG(NH_2_)_0.65_(SH)_0.1_. **(**B) ^13^C NMR spectrum (DMSO-d_6_, 176 MHz) of dPG(NH_2_)_0.75_, dPG(NH_2_)_0.65_(NB)_0.1_ and dPG(NH_2_)_0.65_(SH)_0.1_.

**Figure S1.2.** HSQC (DMSO-d_6_, 700 MHz, ^1^H; 176 MHz, ^13^C) spectrum **(**A) dPG(NH_2_)_0.75_, (B) dPG(NH_2_)_0.65_(NB)_0.1_ and (C) dPG(NH_2_)_0.65_(SH)_0.1_.

**Figure S1.3.** HMBC (DMSO-d_6_, 700 MHz, ^1^H; 176 MHz, ^13^C) spectrum of **(**A) dPG(NH_2_)_0.75_，(B) dPG(NH_2_)_0.65_(NB)_0.1_ and (C) dPG(NH_2_)_0.65_(SH)_0.1_.

**Figure S1.4.** ^1^H NMR spectrum (600 MHz, CDCl_3_) of compound.

**Figure S1.5.**^13^C NMR spectrum (150.9 MHz, CDCl_3_) of compound.

**Figure S1.6.** ^1^H NMR spectrum (600 MHz, D_2_O) of compound.

**Figure S1.7.** ^13^C NMR spectrum (150.9 MHz, CDCl_3_) of compound.

**Figure S1.8.** Standard curve for Ellman’s test (standard sample: L-cysteine, 0, 5, 10, 20, 40, 60, 80, 100 μM), absorbance at 412 nm obtained by Plate reader. A linear regression model was applied to fit the data and generate the corresponding standard curve.

**Figure S1.9.** Absorbance at 412 nm of dPG(NH_2_)_0.65_(SH)_0.1_.

**Table S1.** Thiol group content of different nanogels.

| NGs | Feeding thiol group  (μmol) | Absorbance at  412 nm | Remaining thiol group (μmol) | Consumption thiol group ratio  (%) |
| --- | --- | --- | --- | --- |
| NG_0.80_ | 2.110 | 0.030 ± 0.001 | 0.191 ± 0.079 | 90.949 ± 3.755 |
| NG_0.67_ | 3.480 | 0.032 ± 0.001 | 0.349 ± 0.079 | 89.958 ± 2.277 |
| NG_0.50_ | 5.280 | 0.035 ± 0.001 | 0.614 ± 0.121 | 88.379 ± 2.292 |
| NG_0.33_ | 7.070 | 0.034 ± 0.002 | 0.508 ± 0.238 | 92.816 ± 3.362 |
| NG_0.20_ | 8.440 | 0.040 ± 0.002 | 1.010 ± 0.165 | 88.036 ± 1.954 |

[a] Feeding thiol group: Thiol group added as starting material in the preparation of nanogels. [b] Remaining thiol group: Thiol group remained in Nanogel. [c] Absorbance at 412 nm obtained by Plate reader.

**Table S2.** Amino group content of compounds (%, n = 3)

| dPG_10_(NH_2_)_0.75_ | dPG(NH_2_)_0.65_(NB)_0.1_ | dPG(NH_2_)_0.65_(SH)_0.10_ |
| --- | --- | --- |
| 75.42 ± 0.75 | 63.57 ± 1.12 | 64.12 ± 1.41 |

**Table S3.** Amino group content of NGs (μmol -NH_2_/mg, n = 3)

| NG_0.80_ | NG_0.67_ | NG_0.50_ | NG_0.33_ | NG_0.20_ |
| --- | --- | --- | --- | --- |
| 2.65 ± 0.17 | 2.14 ± 0.24 | 1.85 ± 0.19 | 2.23 ± 0.18 | 2.79 ± 0.20 |

**Table S4.** Amino group content of post functionalization nanogels (μmol -NH_2_/mg, n = 3)

| NG_0.33_-Fuc | NG_0.33_-Gal | PNG_0.33_-Fuc | PNG_0.33_-Gal |
| --- | --- | --- | --- |
| 1.52 ± 0.11 | 1.48 ± 0.09 | 1.21 ± 0.19 | 1.19 ± 0.18 |

**Figure S1.10.** Storage modulus of hydrogels of the same formulation as Nanogel.

**Figure S1.11.** GPC (H_2_O) spectrum of dPG(NH_2_)_0.75_ (3 mg/mL, 1 mL/min).

**Figure S2.** (A) Size and Zeta potential for different crosslinking degrees NG-Fuc (mean ± SD, n = 3), (B) Size and Zeta potential for different crosslinking degrees NG-Gal (mean ± SD, n = 3), (C) Size distribution of PNG_0.33_-Fuc, (D) Size distribution of PNG_0.33_-Gal.

**Figure S3.** (A) Cytotoxicity of different NG-Fuc with L929 cells (mean ± SD, n = 7), (B) Cytotoxicity of different NG-Gal with L929 cells (mean ± SD, n = 7), (C) Live/dead staining of NGs with L929 cells.

**Figure S4.** (A) Cytotoxicity of different NG-Fuc with A549 cells (mean ± SD, n = 7), (B) Cytotoxicity of different NG-Gal with A549 cells (mean ± SD, n = 7), (C) Live/dead staining of NGs with A549 cells.

**Figure S5.** (A) Cytotoxicity of different PNG_0.33_-Fuc with L929 cells (mean ± SD, n = 7), (B) Cytotoxicity of different PNG_0.33_-Gal with L929 cells (mean ± SD, n = 7), (C) Hemolytic of PNG_0.33_-Fuc (mean ± SD, n = 7), (D) Hemolytic of PNG_0.33_-Gal (mean ± SD, n = 7).

**Figure S6.** (A) Co-localization for NGs with *P. aeruginosa*. (Red: ^Rhodamine^Nanogel. Green: ^FITC^*P. aeruginosa*, scar bar = 20 μm), (B) Flow cytometry analysis of NGs co-incubated with planktonic *P. aeruginosa*.

**Figure S7.** (A) OD_600_ value of planktonic *P.a.* after 24h of treatment with NG-Fuc (mean ± SD, n = 7), (B) Planktonic *P.a.* inhibition after 24h of treatment with NG-Fuc on CFUs (mean ± SD, n = 7), (C) Biofilm biomass of *P.a.* after 72h of treatment with NG-Fuc (mean ± SD, n = 7), (D) 48h growth curve after treatment with NG-Fuc.

**Figure S8.** (A) OD_600_ of planktonic *P.a.* after 24h of treatment with NG-Gal (mean ± SD, n = 7), (B) Planktonic *P.a.* inhibition after 24h of treatment with NG-Gal on CFUs (mean ± SD, n = 7), (C) Biofilm biomass of *P.a.* after 72h of treatment with NG-Gal (mean ± SD, n = 7), (D) 48h growth curve after treatment with NG-Gal.

**Figure S9.** P. aeruginosa biofilm formation after 72h of treatment with samples (mean ± SD, n=7).

**Figure S10.** (A) Representative live/dead bacterial staining of biofilm-coating P. aeruginosa after 12h treatment (green: live, red: dead, scale bar = 60 μm) and (B) related Fluorescence Signal Statistics.

**Figure S11.** (A) *P.a.* biofilm formation after treatment with samples (mean ± SD, n = 7), (B) and (C) *P.a.* biofilm inhibition after 24h of treatment with samples (mean ± SD, n = 7), (D) biofilm viability after 12h of treatment with samples (mean ± SD, n = 7).

**Figure S12.** (A) *E. coli* survival ratio after treatment with samples (mean ± SD, n = 7), (B) MRSA survival ratio after treatment with samples (mean ± SD, n = 7), (C) Biofilm eradication for samples with *E. coli* (mean ± SD, n = 7), (D) Biofilm eradication for samples with MRSA (mean ± SD, n = 7), (C) Biofilm viability for samples with *E. coli* (mean ± SD, n = 7), (D) Biofilm viability for samples with MRSA (mean ± SD, n = 7).

# 3. References

[1]	S. Roller, H. Zhou, R. Haag, *Mol. Diversity* **2005**, *9*, 305-316.

[2]	J. R. Romeo, L. McDermott, C. S. Bennett, *Org. Lett.* **2020**, *22*, 3649-3654.

[3]	R. U. Kadam, M. Bergmann, M. Hurley, D. Garg, M. Cacciarini, M. A. Swiderska, C. Nativi, M. Sattler, A. R. Smyth, P. Williams, *Angew. Chem. Int. Ed.* **2011**, *50*, 10631.

[4]	C. J. Capicciotti, R. S. Mancini, T. R. Turner, T. Koyama, M. G. Alteen, M. Doshi, T. Inada, J. P. Acker, R. N. Ben, *ACS Omega* **2016**, *1*, 656-662.

[5]	S. Le Luyer, P. Guégan, N. Illy, *Macromolecules* **2022**, *55*, 5430-5440.

[6]	P. Espeel, F. E. Du Prez, *Eur. Polym. J.* **2015**, *62*, 247-272.

[7]	J. Garel, D. S. Tawfik, *Chemistry–A European Journal* **2006**, *12*, 4144-4152.

[8]	Z. Xu, Y. Liang, S. Lin, D. Chen, B. Li, L. Li, Y. Deng, *Curr. Microbiol.* **2016**, *73*, 474-482.

[9]	J. Lu, L. Turnbull, C. M. Burke, M. Liu, D. A. Carter, R. C. Schlothauer, C. B. Whitchurch, E. J. Harry, *PeerJ* **2014**, *2*, e326.

[10]	S. E. Mountcastle, N. Vyas, V. M. Villapun, S. C. Cox, S. Jabbari, R. L. Sammons, R. M. Shelton, A. D. Walmsley, S. A. Kuehne, *npj Biofilms Microbiomes* **2021**, *7*, 44.

[11]	S. Ravaioli, D. Campoccia, P. Speziale, G. Pietrocola, B. Zatorska, A. Maso, E. Presterl, L. Montanaro, C. R. Arciola, *Biofouling* **2020**, *36*, 86-100.

[12]	Y. Shen, S. Stojicic, M. Haapasalo, *Journal of endodontics* **2010**, *36*, 1820-1823.

[13]	T. Kawahara, M. Takita, A. Masunaga, H. Morita, T. Tsukatani, K. Nakazawa, D. Go, S. Akita, *Int. J. Mol. Sci.* **2019**, *20*, 312.
